# Serum changes in pyridinoline, type II collagen cleavage neoepitope and osteocalcin in early stage male brucellosis patients

**DOI:** 10.1038/s41598-020-72565-8

**Published:** 2020-10-14

**Authors:** Qiang Li, Lansheng Hu, Zhijun Zhao, Li Ma, Jiquan Li, Liqing Xu, Jiming Wang

**Affiliations:** 1 Qinghai Institute for Endemic Disease Prevention and Control, Xining, 811602 Qinghai China; 2 Ruikang Hospital Affiliated to Guangxi University of Chinese Medicine, Nanning, China

**Keywords:** Molecular biology, Biomarkers, Medical research, Rheumatology

## Abstract

Musculoskeletal changes are the most common clinical manifestation of brucellosis. The main objective of this study was to provide a better understanding of this disease, while also attempting to identify potential markers that can identify the early stage musculoskeletal changes associated with human brucellosis. In this case–control study, 41 male early-stage brucellosis patients (within 6 months of diagnosis) who had not received drug therapy and 44 matched controls were examined. Venous blood samples were collected and serum pyridinoline (PYD), type II collagen cleavage neoepitope (C2C) and osteocalcin (OC) levels were quantified using an enzyme-linked immunosorbent assay (ELISA). In the brucellosis group, the median serum levels of PYD (278.53 µg/L), C2C (82.23 µg/L) and OC (8.41 µg/L) were significantly elevated relative to the control group (Z = 5.686, 3.997, 3.579; *P* = 0.000). Serum PYD, C2C, and OC levels were increased in early-stage male brucellosis patients, and these factors appear to have promise as potential indicator biomarkers that can reflect the osteoarticular changes that occur in the early stage of human brucellosis.

## Introduction

Brucellosis is a zoonotic disease that severely impacts livestock productivity and remains a major public health problem in the Mediterranean region, the Middle East, Africa, Latin America, and parts of Asia^1^. In China, brucellosis has been reported in all the 32 provinces and is endemic in humans in 25 of 32 provinces (or autonomous regions) in mainland China^2^. Thus, brucellosis poses a serious threat to public health^3^.

Brucellosis is transmitted to people by direct or indirect contact with infected animals or the consumption of contaminated foods^4^. Clinical presentation of this disease is nonspecific and highly variable and can include fever, headache, chills, myalgia, and arthralgia^5^. Although several organs and organ systems may be involved in this disease, the most common clinical presentations are musculoskeletal, including sacroiliitis, spondylitis, peripheral arthritis, osteomyelitis, discitis, bursitis, and tenosynovitis^6^. As musculoskeletal changes progress, patients tend to initially visit a general practitioner and ultimately consult a rheumatologist and orthopedic specialists. However, due to variable clinical features and the lack of specific symptoms, obtaining a diagnosis and the necessary drug therapy tends to be delayed^7^, making an early diagnosis imperative to minimizing the musculoskeletal changes associated with brucellosis.

In human brucellosis, it would seem that abnormal musculoskeletal changes are present during the early stage despite changes being undetectable via radiography. While a few studies have identified serum cartilage oligomeric matrix protein (COMP) and C-terminal telopeptide of type II collagen (CTX-II) as biomarkers that are associated with brucellosis-induced musculoskeletal changes^11,12^, there is still a need to identify potential bone markers that can identify early changes and aid in an early diagnosis. When assessing osteoarthritis (OA), commonly utilized biomarkers include pyridinoline (PYD), type II collagen cleavage neoepitope (C2C), and osteocalcin (OC). PYD has been validated as a useful marker for bone resorption, with significantly higher levels seen in OA patients^8^. Furthermore, C2C levels have been associated with cartilage degradation^9^, whereas OC levels reflect osteoblastic bone formation and mineralization levels^10^. Thus, this study examined PYD, C2C, and OC in serum obtained from human brucellosis patients for the first time in an attempt to determine if they may serve as potential biomarkers able to identify early-stage brucellosis-induced musculoskeletal changes. It is hoped that these findings will contribute to a better understanding of this disease.

## Materials and methods

### Patients and sample processing

In this case–control study, serum samples were collected at the Qinghai Institute for Endemic Disease Prevention and Control (Qinghai, China) from 2017 and 2018. Each patient was examined serologically and by X-ray imaging, and any subjects with a joint disease, such as rheumatic fever, osteoarthritis, rheumatic arthritis, paratyphoid fever, or tuberculosis, were excluded as these joint diseases have similar clinical manifestations to human brucellosis. Patients were determined brucellosis positive following clinical examination, serological examination, and epidemiological examination, and 41 male patients who were within 6 months of receiving a diagnosis and had not received drug therapy were selected. For some of these patients, a definite time of infection was unclear, so acute and subacute patients were also defined as at the early period of brucellosis. Clinically, of some of the brucellosis patients presented with fever, weakness, and headache, whereas others had no clear symptoms, but were examined due to their increased chance of occupational exposure or living environment, both. Additionally, 44 matched male control samples were also obtained and had similar occupational risks or living environments.

Serum samples from fasting blood were collected and stored at − 80 °C until assayed. Serum levels of PYD, C2C, and OC were detected using enzyme-linked immunosorbent assay (ELISA) kits (Beijing Air-hx biological technology Co. Ltd., Beijing, China) according to the manufacturer’s protocols(the detecting ranges were 1.0–200 nmol/L, 1.0–80 μg/L, 0.1–8 μg/L, respectively, and intra‐ and inter-assay CVs were less than 9% and 15%). This study was performed in compliance with the ethical principles in the Declaration of Helsinki and was approved by the ethics committee of the Qinghai Institute for Endemic Disease Prevention and Control. All subjects provided informed consent.

### Brucellosis diagnostic criteria

Patients were diagnosed based on Chinese brucellosis diagnosis criteria (WS 269-2007). The brucellosis patients were divided into three groups, including acute (< 3 months post-infection, high serum titer (≥ 1:100), and high fever), subacute (3–6 months post-infection, positive serum titer (≥ 1:100), and low fever) and chronic (> 6 months post-infection, positive serum titer, and normal body temperature). The predominant clinical manifestations were fever, weakness, headache, hyperhidrosis, and joint and muscle aches. Serological tests were also utilized to establish disease, including the rose bengal plate test (RBPT, a primary screen test) and a serum agglutination test (SAT, titer ≥ 1:100), meanwhile, SAT is a confirmation test with high sensitivity and high specificity The matched controls tested negative for these serological tests.

#### Exclude criteria

At first, all the study population were excluded normal joint diseases, such as rheumatic fever, osteoarthritis, rheumatic arthritis, paratyphoid fever, and tuberculosis which these joint diseases were similar to human brucellosis at the clinical manifestation .Then, we asked the disease history for each subjects to exclude thyroid dysfuctions, chronic liver and/or kidney diseases, Ehlers–Danlos Syndrome and malignancies with bone metastasis.

### Statistical analysis

Data were analyzed using SPSS 17.0 software (SPSS, Chicago, IL, USA). The data was analyzed using a *t*-test and data are displayed as a mean ± standard, with *P* < 0.05 deemed significant. The data for PYD, C2C, and OC expression levels were nonparametric and thus analyzed using a Wilcoxon rank-sum test.

### Consent for publication

All of the authors have read the manuscript and have agreed to submit it in its current form for consideration for publication.

## Results

According to epidemiological data, clinical expression and serological test (RBPT positive and SAT titer ≥ 1:100), all the patients were diagnosed as early patients of brucellosis. The average age of the brucellosis patients was 39.69 ± 9.98 years and for the control group was 42.07 ± 13.70 years, with no significant difference in age between the groups (*t* = 0.912, *P* = 0.364). When examining serum PYD, C2C, and OC levels, the median levels in the brucellosis group were 278.53 nmol/L, 82.23 µg/L, and 8.41 µg/L, respectively, whereas in the control group, the median levels were 210.54 nmol/L, 72.74 µg/L, and 7.43 µg/L, respectively. When comparing the levels in both groups using a Wilcoxon rank-sum test, the PYD (Z = 5.686), C2C (Z = 3.997), and OC (Z = 3.579) levels were all significantly different between the two groups (*P* = 0.000, Table 1).

**Table 1:** Serum PYD, C2C and OC levels.

Biomarkers	Patients				Healthy control				Z	*P*
	Median	Range	25% IRQ	75% IRQ	Median	Range	25% IRQ	75% IRQ		
PYD (nmol/L)	278.53	139.58–397.32	233.07	314.72	210.54	86.61–244.89	191.38	224.46	5.686	0.000
C2C (µg/L)	82.23	46.85–111.12	75.33	92.17	72.74	29.61–94.35	59.28	92.17	3.997	0.000
OC (µg/L)	8.41	3.33–17.19	7.93	9.09	7.43	2.69–11.42	6.93	8.18	3.579	0.000

*IRQ* interquartile range.

## Discussion

Human brucellosis can cause serious complications and obtaining an early diagnosis is very difficult and is often met with delays or even misdiagnoses^7,13^. Furthermore, while radiography is a useful method for diagnosing brucellosis-induced musculoskeletal changes, it is only able to provide a low level of sensitivity during the early stages; thus, relying on radiography can often delay a diagnosis^14^. The brucellosis-induced musculoskeletal changes are frequently destructive and associated with osteopenia and cartilage damage^15^. Components that have been identified as associated with brucellosis include metalloproteinases, which may be promising indicators for determining osteoarticular changes^16^, and gamma interferon (IFN-γ) and tumor necrosis factor alpha (TNF-α), which are involved in brucellosis pathophysiology and inflammatory activation^17^. However, these biomarkers are not specific and cannot provide a definitive clinical diagnosis. So, it is necessary to find biomarkers that reliably reflect the musculoskeletal changes associated with human brucellosis.

PYD is derived from collagen breakdown and has been shown to be an indicator of bone resorption in OA patients^8^. Furthermore, urinary PYD was found to be significantly correlated with radiographic OA grades and associated with the synthesis of osteophytes, subchondral bone sclerosis and synovial degeneration, as well as cartilage degeneration in the joints^18^. C2C is a type II collagen degradation product (3/4 fragment) that appears to reflect the extent of cartilage degradation in OA patients^9^. C2C has also been shown to correlate with knee degeneration in patients with symptomatic knee osteoarthritis^19^. Moreover, OC is one of the most abundant non-collagen bone proteins in the bone matrix and has been used as a biomarker for monitoring bone formation and bone turnover^20^, with its synthesis significantly increased during the remodeling processes^21^. In this study, the PYD, C2C, and OC serum levels were all statistically elevated in brucellosis individuals relative to the healthy controls, and their elevation indicates abnormal changes in the collagen metabolism in cartilage, bone formation, and bone turnover.

Brucellosis more commonly affects males rather than females^22^, with a ratio of 5:2–5:3 in endemic areas^13^. It is possible that the increased prevalence in male patients reflects the exposure pattern of human brucellosis. Furthermore, male patients predominately experience spinal brucellosis, which is the foremost cause of the debilitating and disabling complications of brucellosis^23^. Thus, PYD, C2C, and OC serum changes may be useful in providing an early brucellosis diagnosis in males, but to make these factors useful markers in females, the effects of osteoporosis due to menopause and age would have to be ruled out.

Meanwhile, there were a few limitations in study. Firstly , there are many types of osteoarticular changes among brucellosis patients, and, some of brucellosis patients at an early stage do not have imaging changes of osteoarticular changes, so, this study only have laboratory investigations without radiological evidence. Secondly, these biomarkers can reflect not each type of osteoarticular changes. Thus, future research should focus on comparing PYD, C2C, and OC changes in individuals with brucellosis, osteoarthritis, and osteoarthritis/brucellosis to determine the true specificity of these markers. Furthermore, the study set should be expanded in future studies as abnormal changes could happen at early stages and knowing how these proteins change over time would be insightful. Also, a larger study set can help confirm the findings presented here.

## Conclusion

In this study, PYD, C2C, and OC serum levels were increased in male brucellosis patients at an early stage. These factors are associated with abnormal changes in the collagen metabolism in cartilage, bone formation, and bone turnover. These findings suggest that these factors may potentially serve as indicator biomarkers for osteoarticular changes that occur during the early stage of human brucellosis. Future research should focus on examining the relationship between these biomarkers and early-stage brucellosis patients with/without osteoarticular clinical symptoms.

## Data Availability

All the data is contained within the manuscript.

## References

[1] Pappas G, Papadimitriou P, Akritidis N, Christou L, Tsianos EV (2006). The new global map of human brucellosis. Lancet Infect. Dis..

[2] Shang D, Xiao D, Yin J (2002). Epidemiology and control of brucellosis in China. Vet. Microbiol..

[3] Zhang X, Wang Z, Mu G, Wang T (2015). Brucellosis control in northeast China: a long way to go. Public Health.

[4] Lai S, Zhou H, Xiong W, Gilbert M, Huang Z, Yu J (2017). Changing epidemiology of human brucellosis, China, 1955–2014. Emerg. Infect. Dis..

[5] Njeru J, Wareth G, Melzer F, Henning K, Pletz MW (2016). Systematic review of brucellosis in Kenya: disease frequency in humans and animals and risk factors for human infection. BMC Public Health.

[6] Shi Y, Gao H, Pappas G, Chen Q, Li M, Xu J (2018). Clinical features of 2041 human brucellosis cases in China. PLoS ONE.

[7] Esmaeilnejad-Ganji SM, Esmaeilnejad-Ganji SMR (2019). Osteoarticular manifestations of human brucellosis: a review. World J. Orthop..

[8] Zhan ZW, Yamamoto I, Morita R (1999). Urinary pyridinoline and deoxypyridinoline as bone metabolic markers in predicting therapeutic effects of estrogen and alfacalcidol in women with osteoporosis. J. Bone Miner. Metab..

[9] Cahue S, Sharma L, Dunlop D, Ionescu M, Song J, Lobanok T (2007). The ratio of type II collagen breakdown to synthesis and its relationship with the progression of knee osteoarthritis. Osteoarthr. Cartil..

[10] Zoch ML, Clemens TL, Riddle RC (2016). New insights into the biology of osteocalcin. Bone.

[11] Zhao ZJ, Li Q, Ma L, Li JQ, Xu LQ (2018). The early diagnostic value of serum neopterin and cartilage oligomeric matrix protein for osteoarticular changes among brucellosis patients at an early period. J. Orthop. Surg. Res..

[12] Zhao ZJ, Li Q, Zhou X, Ma L, Xu LQ, Yang PZ (2016). A primary investigation on serum CTX-II changes in patients infected with brucellosis in Qinghai plateau, China. Biomed. Environ. Sci..

[13] Hashemi SH, Keramat F, Ranjbar M, Mamani M, Farzam A, Jamal-Omidi S (2007). Osteoarticular complications of brucellosis in Hamedan, an endemic area in the west of Iran. Int. J. Infect. Dis..

[14] Mouna CB, Mohamed FL, Mohamed C (2008). Spinal brucellosis: a review. Skelet. Radiol..

[15] Geyik MF, Gür A, Nas K, Cevik R, Saraç J, Dikici B (2002). Musculoskeletal involvement in brucellosis in different age groups: a study of 195 cases. Swiss Med. Wkly..

[16] Šiširak M, Hukić M (2015). Osteoarticular complications of brucellosis: the diagnostic value and importance of detection matrix metalloproteinases. Acta Med. Acad..

[17] Demirdag K, Ozden M, Kalkan A, Godekmerdan A, Kili SS (2003). Serum cytokine levels in patients with acute brucellosis and their relation to the traditional inflammatory markers. FEMS Immunol. Med. Microbiol..

[18] Takahashi M, Naito K, Abe M, Sawada T, Nagano A (2004). Relationship between radiographic grading of osteoarthritis and the biochemical markers for arthritis in knee osteoarthritis. Arthritis Res. Ther..

[19] King KB, Lindsey CT, Dunn TC (2004). A study of the relationship between molecular biomarkers of joint degeneration and the magnetic resonance-measured characteristics of cartilage in 16 symptomatic knees. Magn. Reson. Imaging.

[20] Fairney A, Patel KV, Hollings NP, Seifert MH (1990). Abnormal osteocalcin binding in rheumatoid arthritis. Ann. Rheum. Dis..

[21] Gabusi E, Paolella F, Manferdini C, Gambari L, Kon E, Filardo G (2018). Cartilage and bone serum biomarkers as novel tools for monitoring knee osteochondritis dissecans treated with osteochondral scaffold. BioMed Res. Int..

[22] Tsolia M, Drakonaki S, Messaritaki A (2002). Clinical features, complications and treatment outcome of childhood brucellosis in central Greece. J. Infect..

[23] Liang C, Wei W, Liang X, De E, Zheng B (2019). Spinal brucellosis in Hulunbuir, China, 2011–2016. Infect. Drug Resist..

